# Commentary: Augmented reality in neurosurgery, state of art and future projections. A systematic review

**DOI:** 10.3389/fsurg.2023.1218308

**Published:** 2023-06-30

**Authors:** Andrew Willett, Mohammad Haq, Joseph Holland, Elizabeth Bridwell

**Affiliations:** University of Louisville School of Medicine, Center for Health Process and Innovation, Bluegrass Biodesign, Louisville, KY, United States

**Keywords:** augmented (virtual) reality, neurosurgery, pedicle screw accuracy, neuronavigation, neurooncology

**A Commentary on:**
Augmented Reality in Neurosurgery, State of Art and Future Projections. A Systematic Review By Cannizzaro D, Zaed I, Safa A, Jelmoni AJM, Composto A, Bisoglio A, Schmeizer K, Becker AC, Pizzi A, Cardia A, Servadei F. (2022). Front Surg. 9:864792. doi: 10.3389/fsurg.2022.864792

Innovation and medicine are inseparable, and technologies such as augmented reality (AR) may transform the modern neurosurgical armamentarium. Per insights shared by Cannizzaro and colleagues in their review article “*Augmented Reality in Neurosurgery, State of Art and Future Projections. A Systematic Review*” (1), AR-assisted neurosurgery is a promising, albeit complex and challenging advancement. Over the past year our team of medical students has engaged in a biomedical innovation curriculum offered by a select cohort of accredited medical schools throughout the United States. We were tasked with evaluating the future of AR in neurosurgery. Thanks to our immersive experience with this unique technology, we felt inclined to comment on this article and share our perspectives in hopes that others will engage in these conversations and further promote AR in medicine, specifically within the field of neurosurgery. We found the review article to be robust and believe that it warrants a significant amount of consideration.

The authors highlight how AR has been largely investigated in spine surgeries, composing 18.2% of their literature review (1). This neurosurgical subspeciality has grown in minimally invasive techniques, a process that has been amplified with the utilization of neuronavigation systems. Studies suggest that AR-assisted pedicle screw placement is legitimate, with some reports sharing 100% accuracy (2) while others share 97.8%–98.5% accuracy (3). Although these numbers are encouraging, we must thoroughly question how this technology challenges more traditional surgical techniques. Why invest in AR if it offers no *significant* benefit? Dennler et al. (4) show that supplemental anatomical information provided via AR may help novice surgeons match the efficacy of expert surgeons with pedicle screw placement. However, to our knowledge, no large-scale randomized control trials to date have compared AR assisted pedicle screw placement vs. traditional pedicle screw placement. Moreover, we do not thoroughly understand if AR-assisted surgeries are cost-effective. We can glean insights from literature that compares the use of free hand techniques with robotic devices; although robotics may have more accuracy in placing pedicle screws and can help decrease postoperative complications, the initial costs (which can approach upwards of $850,000) and increased OR time are thought to drastically outweigh its current benefits (5). Contrastingly, AR systems like Xvision have comparable profiles of improved accuracy but offer lower upfront cost (6), suggesting an opportunity for feasible integration. Other groups must not only reinforce the efficacy and practicality of AR, but also clearly analyze the fiscality of these technologies if veteran and novice surgeons alike are to adopt a new way of operating.

AR is an arguably more alluring tool for neurosurgical oncologists and neurosurgeons working in remote areas seeking to collaborate with distant colleagues, which is a realm we would have appreciated for Cannizzaro and colleagues to further explore. Our review of the available AR neuro-oncology literature has been exciting—particularly, a statistically significant improvement in percent of complete glioma resection in a test group (69.6%) compared to the control group (36.4%) (*p* < .01) has been reported (7). Other studies acknowledge the postoperative improvements associated with AR guided surgeries as patients who underwent AR-assisted tumor resection experienced shorter length of hospital stay and improved postoperative quality of life in comparison to non-AR guided resections (8). Moving forward, AR enthusiasts should emphasize a need to attend to extraoperative components affiliated with AR surgeries—if patients spend less time in the hospital and report higher quality of life following AR-guided surgery, then investing in these technologies becomes clearer. Thus, if we can better pinpoint where AR aligns with the needs of both the surgeon and patient, then integration can be met with less resistance.

Additionally, neurosurgical education is a valuable avenue to pursue, explaining why this field composed 18.2% of the authors' literature review (1). Rather than focusing on the traditional sense of education (i.e., training residents, revamping models of surgical instruction, etc.), developers in AR neurosurgery should emphasize how this technology enhances collaboration among neurosurgeons. AR expedites consultations between remote neurosurgeons, encouraging long-distance conferences regarding complex cases in real time (9). Accordingly, current platforms such as virtual interactive presence and augmented reality (VIPAR) place a surgeon in front of a stereoscopic display capable of remote interaction with a workstation situated in a different region or country (10, 11). The surgeon can share their insights on the respective cases and address gaps in access to care. What we find particularly intriguing about this utilization of AR in neurosurgery is that it does not drastically change workflow, which will encourage acceptance within the field.

Nonetheless, as Cannizzaro and colleagues (1) note, there are multiple obstacles that AR must overcome before diffuse integration into the neurosurgical operating room. There are two points we want to highlight from their speculations: (1) as AR will presumably have a role in the display of intraoperative images, there will be a growing need for strong microcomputers that facilitate access to data without compromising quality and (2) AR surgical headsets and hardware will need to be widely distributable, comfortable, non-obstructive, yet customizable to individual needs (1). We too find these concepts paramount to the future of virtually assisted surgeries. If AR can accurately augment intraoperative neuroimaging, it has the potential to significantly decrease the number of times a neurosurgeon must look away from the operating field, diminishing and possibly eliminating the need to constantly reorient from the patient to a distant screen in the operating room and back (12). Although some technologies allow for this, they are quite cumbersome for the surgeon, requiring them to wear a large, bulky headset (13). To further complicate matters, currently available AR technologies may not fully integrate with a surgeon's established hospital, software, and/or current neuronavigation systems, which makes a future with AR burdensome and laborious.

Naturally, these conversations will spark excitement and criticism, but we cannot let this outweigh the need to continue expanding and diversifying the future of medicine. We thank Cannizzaro and colleagues (1) for their thoughtful contributions to the current discourse and are excited to see how AR and neurosurgery will co-evolve.

## References

[1] Cannizzaro D Zaed I Safa A Jelmoni AJM Composto A Bisoglio A Augmented reality in neurosurgery, state of art and future projections. A systematic review. Front Surg. (2022) 9:864792. 10.3389/fsurg.2022.864792 35360432 PMC8961734

[2] Yahanda AT Moore E Ray WZ Pennicooke B Jennings JW Molina CA. First in-human report of the clinical accuracy of thoracolumbar percutaneous pedicle screw placement using augmented reality guidance. Neurosurg Focus. (2021) 51(2):E10. 10.3171/2021.5.FOCUS21217 34333484

[3] Liu A Jin Y Co6rill E Khan M Westbroek E Ehresman J Clinical accuracy and initial experience with augmented reality-assisted pedicle screw placement: the first 205 screws. J Neurosurg Spine. (2021) 36(3):1–7. 10.3171/2021.2.SPINE202097 34624854

[4] Dennler C Jaberg L Spirig J Agten C Gotschi T Furnstahl P Augmented reality-based navigation increases precision of pedicle screw insertion. J Orthop Surg Res. (2020) 15(1):174. 10.1186/s13018-020-01690-x 32410636 PMC7227090

[5] Ezeokoli EU Pfennig M John J Gupta R Khalil JG Park DK. Index surgery cost of fluoroscopic freehand versus robotic-assisted pedicle screw placement in lumbar instrumentation: an age, sex, and approach-matched cohort comparison. J Am Acad Orthop Surg Glob Res Rev. (2022) 6(12): 3–6. 10.5435/JAAOSGlobal-D-22-00137 PMC9722569 36732310

[6] Rush AJ III Shepard N Nolte M Siemionow K Phillips F. Augmented reality in spine surgery: current state of the art. Int J Spine Surg. (2022) 16(S2):S22–7. 10.14444/8273 36266050 PMC9808789

[7] Sun GC Wang F Chen XL Yu XG Ma XD Zhou DB Impact of virtual and augmented reality based on intraoperative magnetic resonance imaging and functional neuronavigation in glioma surgery involving eloquent areas. World Neurosurg. (2016) 96:375–82. 10.1016/j.wneu.2016.07.107 27521727

[8] Yang DL Xu QW Che XM Wu JS Sun B. Clinical evaluation and follow-up outcome of presurgical plan by dextroscope: a prospective controlled study in patients with skull base tumors. Surg Neurol. (2009) 72(6):682–9; discussion 689. 10.1016/j.surneu.2009.07.040 19850330

[9] Kockro RA Stadie A Schwandt E Reisch R Charalampaki C Ng I A collaborative virtual reality environment for neurosurgical planning and training. Neurosurgery. (2007) 61(5 Suppl 2):379–91; discussion 391. 10.1227/01.neu.0000303997.12645.26 18091253

[10] Shenai MB Dillavou M Shum C Ross D Tubbs RS Shih A Virtual interactive presence and augmented reality (VIPAR) for remote surgical assistance. Neurosurgery. (2011) 68(Suppl 1):200–7; discussion 207. 10.1227/NEU.0b013e3182077efd 21304333

[11] Davis MC Can DD Pindrik J Rocque BG Johnston JM. Virtual interactive presence in global surgical education: international collaboration through augmented reality. World Neurosurg. (2016) 86:103–11. 10.1016/j.wneu.2015.08.053 26342783 PMC5476961

[12] Matsukawa K Yato Y. Smart glasses display device for fluoroscopically guided minimally invasive spinal instrumentation surgery: a preliminary study. J Neurosurg Spine. (2020) 34(1):1–6. 10.3171/2020.6.SPINE20644 33049696

[13] Xvision: the future of surgery is within sight. Available at: https://augmedics.com

